# Comprehensive Analysis of the Complete Mitochondrial Genomes of *Dendrobium nobile* Lindl. and *Dendrobium denneanum* Kerr., Two Precious Traditional Chinese Medicinal Herbs

**DOI:** 10.3390/ijms27083441

**Published:** 2026-04-11

**Authors:** Tao He, Leyi Zhao, Xiaoli Fan, Tianfang Huang, Yanling Jin, Zhuolin Yi, Yongqiang Liu, Yu Gao, Hai Zhao

**Affiliations:** 1 Chengdu Institute of Biology, Chinese Academy of Sciences, Chengdu 610213, China; fanxl@cib.ac.cn (X.F.); huangtf@cib.ac.cn (T.H.); jinyl@cib.ac.cn (Y.J.); yizl@cib.ac.cn (Z.Y.); liuyq0129@163.com (Y.L.); gaoyu211@mails.ucas.ac.cn (Y.G.); 2 Department of Natural Sciences, Pitzer College, Claremont, CA 91711, USA; lzhao@students.pitzer.edu

**Keywords:** *Dendrobium nobile* Lindl., *Dendrobium denneanum* Kerr., mitochondrial genome, multi–chromosome circular structure, intracellular gene transfer

## Abstract

The plant mitochondrial genome has become a current research hotspot as an independent genetic model. Nevertheless, mitochondrial genome information for most *Dendrobium* species remains unknown. In this study, the assembly of mitochondrial genome of *Dendrobium nobile* Lindl.,1830 and *Dendrobium denneanum* Kerr., 1933 was conducted through the application of second- and third-generation sequencing technologies, with the mitochondrial genome of *D. denneanum* Kerr. being reported first. The results revealed that the mitochondrial genomes of the two species possessed a multi-chromosome circular structure. Their total lengths were 641,414 bp and 558,760 bp, consisting of 21 and 19 contigs, respectively. A total of 67 and 72 genes, 993 and 1491 repeat sequences, and 549 and 553 RNA editing sites were identified. Gene loss was observed. A total of 26 and 36 homologous fragments were detected between the mitochondrial and the chloroplast genome, accounting for 5.09% and 4.93% of the total lengths, respectively, indicating intracellular gene transfer. Synteny and phylogenetic analyses revealed that the two species shared extensive collinear regions and clustered together in a distinct clade of the phylogenetic tree, indicating a close sister relationship. These findings enrich the mitochondrial genome database and provide valuable insights to guide future research on species identification and molecular evolution of the genus *Dendrobium*.

## 1. Introduction

*Dendrobium nobile* Lindl. and *D. denneanum* Kerr. are two *Dendrobium* species in the Orchidaceae family; both are precious traditional Chinese medicinal herbs and are listed in the Pharmacopoeia of the People’s Republic of China [1]. The two species are used as raw materials for producing health products that have special efficacy in moistening the lungs, generating body fluid, nourishing yin and clearing heat, owing to some important chemical contents, including alkaloids, polysaccharides, flavonoids, tannins, etc. Meanwhile, their blooms are very beautiful, with vibrant colors. Since the wild resources of the two species are protected under national key protection, the current strategy is to develop artificial cultivation, which plays a role in windbreaking, soil stabilization, and beautifying barren mountains. Given their significant medicinal, ecological, and scientific value, our previous studies have focused on various aspects of the two species, including at morphological, chemical, and molecular levels [2,3,4,5,6,7,8,9]. At the molecular level, these studies have involved partial nuclear DNA sequences and partial chloroplast DNA sequences. As research advances, it is necessary to explore other DNA systems in order to accumulate more genetic information.

The analysis of plant mitochondrial genomes has become a current research hotspot as an independent genetic model separate from the nuclear genome. Mitochondria are the genetic system for cellular respiration, supplying energy for various cellular activities and playing a crucial role in plant growth and development [10]. In the early 1860s, genetic material within mitochondria was first discovered [11]. Subsequently, researchers successively identified all the components required for replication, transcription, and protein translation in mitochondria, such as DNA polymerase, RNA polymerase, transfer RNA, ribosomal RNA, and so on. These findings collectively demonstrated that mitochondria possess a relatively independent genetic and transcriptional system. Since the completion of *Arabidopsis thaliana* mitochondrial genome sequencing in 1997 [12], an increasing number of plant mitochondrial genomes have been sequenced and listed in the NCBI-Genome database with the development and widespread adoption of high-throughput sequencing technologies in recent years [13]. The plant mitochondrial genome has also become an important tool for genetic breeding, species identification and population phylogeny [14,15,16].

Within the genus *Dendrobium* (Orchidaceae), the mitochondrial genomes of several species have been reported [17,18,19,20]. These studies showed that the mitochondrial genome of the genus *Dendrobium* has a different structure, including monocyclic and polycyclic structural variation, frequent gene recombination, abundant repetitive sequences, and intracellular gene transfer (IGT). The multi-chromosomal structure provided new insights for the precise identification of medicinal plants [21]. The presence of extensive homologous sequences and repetitive sequences was considered an important reason for genome expansion. However, among the 1500 *Dendrobium* species, fewer than 10 have been sequenced, which is far from sufficient. Mitochondrial genome information for most species remains unknown. Furthermore, even within the same genus and species, differences in the mitochondrial genome may exist due to variations in geographical origin, which has hindered further research. It is necessary to increase the number of samples and conduct more sequencing.

In this study, *D. nobile* Lindl. and *D. denneanum* Kerr., representative species from the Sichuan and Guizhou provinces in southwestern China, were selected as experimental accessions. Firstly, whole-genome sequencing, assembly, and annotation of the mitochondrial genomes of the two species were completed through second- and third-generation sequencing methods. Secondly, codon usage, repeat sequences, RNA editing sites, Ka/Ks value, and homologous sequences between chloroplast and mitochondria were determined and synteny and phylogenetic analysis were conducted. Taken together, we hope these findings will provide valuable insights to guide future research on *Dendrobium* species, expand the genus mitogenome database, and offer important information on genetic variation, species identification, breeding, and molecular evolution within the genus.

## 2. Results

### 2.1. Mitochondrial Genome Characteristics of D. nobile Lindl. and D. denneanum Kerr.

**Figure 1:**
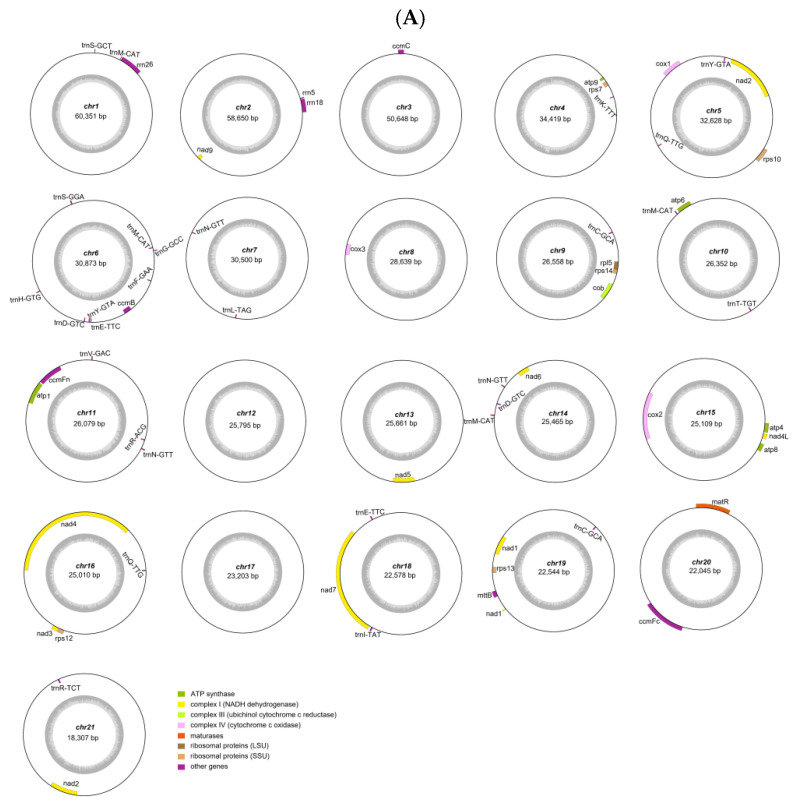

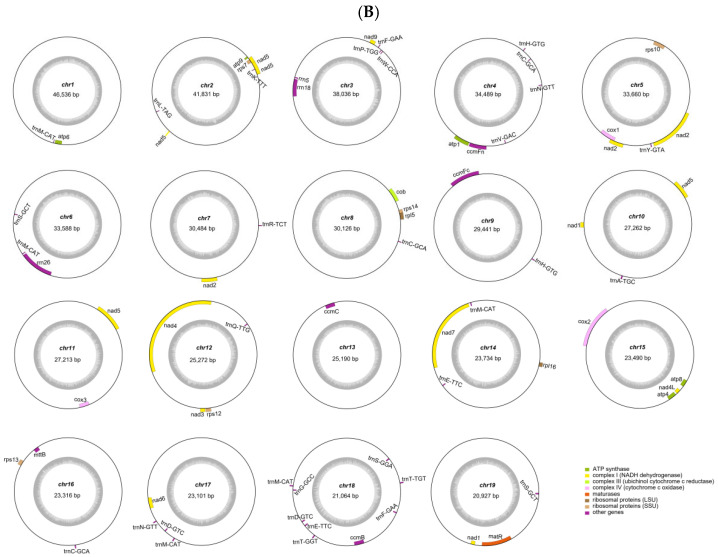
The mitochondrial genome maps of *D. nobile* Lindl. (**A**) and *D. denneanum* Kerr. (**B**). The genome consisted of 21 circular contigs in *D. nobile* Lindl. and 19 circular contigs in *D. denneanum* Kerr.. Each concentric circle represents a circular chromosome sequence. The text in the center indicates the chromosome’s number and length. Genes are classified into different groups based on different colors. Circles outside genes represent forward coding of genes; circles inside genes represent reverse coding of genes. Internal gray circles represent different GC contents.

Illumina and Nanopore sequencing platforms were used to acquire basic data for the mitochondrial genomes of *D. nobile* Lindl. and *D. denneanum* Kerr. Among them, the raw Illumina data were 11.92 Gb and 18.16 Gb, and the Nanopore raw data were 13.04 Gb and 12.57 Gb, with N50 values of 22,850 bp and 23,931 bp and average read lengths of 10,574 bp and 8081 bp, respectively (Supplementary Table S1). Graphical assembly by aligning Illumina and Nanopore sequencing yielded 21 circular contigs for *D. nobile* Lindl. and 19 circular contigs for *D. denneanum* Kerr. (Figure 1), which depict the complete mitochondrial genomes of the two species. We designated these contigs as chromosomes, all of which had a circular structure. Additionally, the total lengths of the mitochondrial genomes were 641,414 bp and 558,760 bp, ranging from 18,307 bp to 60,351 bp and 20,927 bp to 46,536 bp, respectively. The average GC content was 43.40% and 43.49%, with ranges of 39.64% to 46.18% and 37.99% to 45.07%, respectively (Table 1).

The complete mitochondrial genomes of *D. nobile* Lindl. and *D. denneanum* Kerr. were annotated, containing a total of 67 and 72 genes, respectively, including 35 and 36 protein-coding genes (PCGs), 29 and 33 tRNA genes, and three rRNA genes (Supplementary Table S2). On the one hand, the two species shared the same PCGs, including ATP synthase (*atp1*, *atp4*, *atp6*, *atp8*, *atp9*), cytochrome c biogenesis (*ccmB*, *ccmC*, *ccmFc*, *ccmFn*), ubiquinol cytochrome c reductase (*cob*), cytochrome c oxidase (*cox1*, *cox2*, *cox3*), maturases (*matR*), transport membrane protein (*mttB*), NADH dehydrogenase (*nad1*, *nad2*, *nad3*, *nad4*, *nad4L*, *nad5*, *nad6*, *nad7*, *nad9*), and small subunits of ribosomes (SSUs) (*rps10*, *rps12*, *rps13*, *rps14*, *rps7*). The ribosomal RNAs included *rrn18*, *rrn26*, and *rrn5*. Among the PCGs, both species also had the same number of introns: *ccmFc* had one intron, *cox2* had two introns, *nad4* contained three introns, and *nad1*, *nad2*, *nad5* and *nad7* each contained four introns. On the other hand, the difference was that *D. nobile* Lindl. had only one large subunit of a ribosome (*rpl5*), while *D. denneanum* Kerr. had two large subunits of ribosomes (*rpl16*, *rpl5*). Simultaneously, while some tRNA genes were the same in the two species *(trnC-GCA*, *trnD-GTC*, *trnE-TTC*, *trnF-GAA*, *trnG-GCC*, *trnH-GTG*, *trnK-TTT*, *trnL-TAG*, *trnM-CAT*, *trnN-GTT*, *trnQ-TTG*, *trnR-ACG*, *trnR-TCT*, *trnS-GCT*, *trnS-GGA*, *trnT-TGT*, *trnV-GAC*, *trnY-GTA*), only *D. nobile* Lindl. had *trnI-TAT* and *D. denneanum* Kerr. possessed three additional tRNA genes: *trnA-TGC*, *trnP-TGG*, and *trnW-CCA* (Table 2).

### 2.2. RNA Editing Sites of Mitochondrial Genomes of D. nobile Lindl. and D. denneanum Kerr.

**Figure 2:**
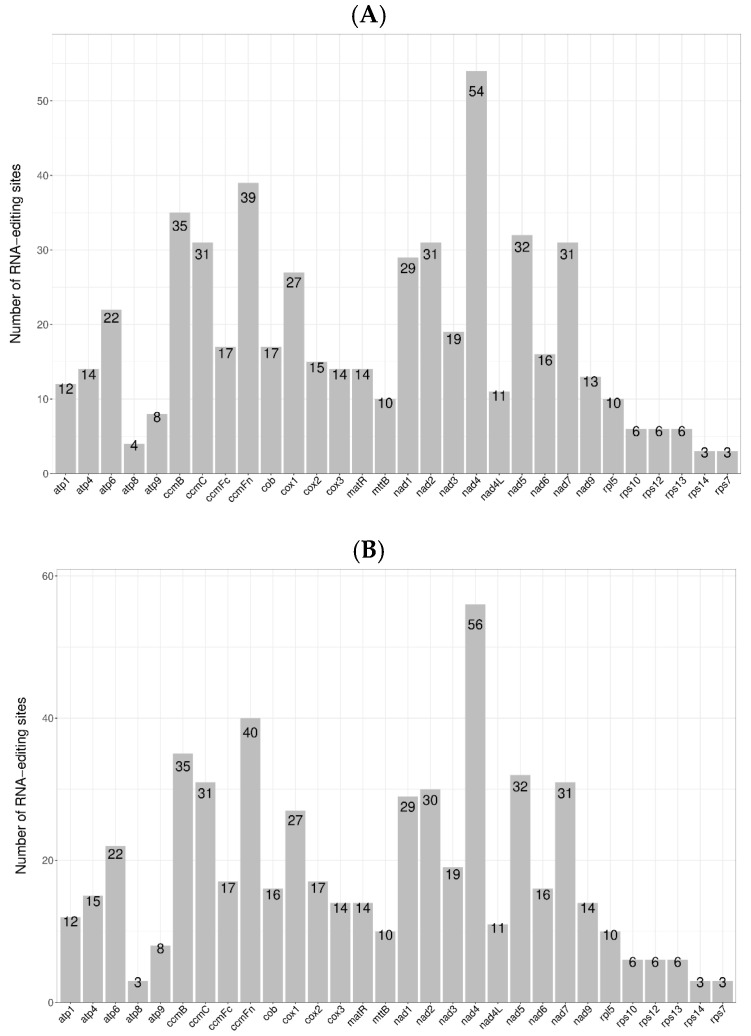
Statistics of the number of RNA editing sites for each gene of *D. nobile* Lindl. (**A**) and *D. denneanum* Kerr. (**B**). The *x*-axis represents the gene; the *y*-axis represents the quantity.

In the mitochondrial genomes of *D. nobile* Lindl. and *D. denneanum* Kerr., a total of 549 and 553 RNA editing sites, respectively, were predicted in PCGs (Figure 2). The distribution of these editing sites across different genes was uneven, ranging from 3 to 54 or 56 sites. Among these PCGs, the *nad4* gene had the most RNA editing sites, with 54 and 56 sites, accounting for 9.84% and 10.13% respectively; it was followed by the *ccmFn* gene, which had 39 and 40 RNA editing sites, accounting for 7.10% in both species. The genes with the fewest RNA editing sites were *rps14* and *rps7*, each having only three editing sites. Furthermore, after RNA editing, the hydrophilicity or hydrophobicity of the amino acids encoded by the codons may change. Further analysis showed that 264 and 266 RNA editing sites (48.09%, 48.10%) led to amino acids changing from hydrophilic to hydrophobic and 50 sites (9.11%, 9.04%) from hydrophobic to hydrophilic; only two editing sites (0.36%) of the amino acids became stop codons (Table 3).

### 2.3. Relative Synonymous Codon Usage (RSCU) of the Mitochondrial Genomes of D. nobile Lindl. and D. denneanum Kerr.

**Figure 3:**
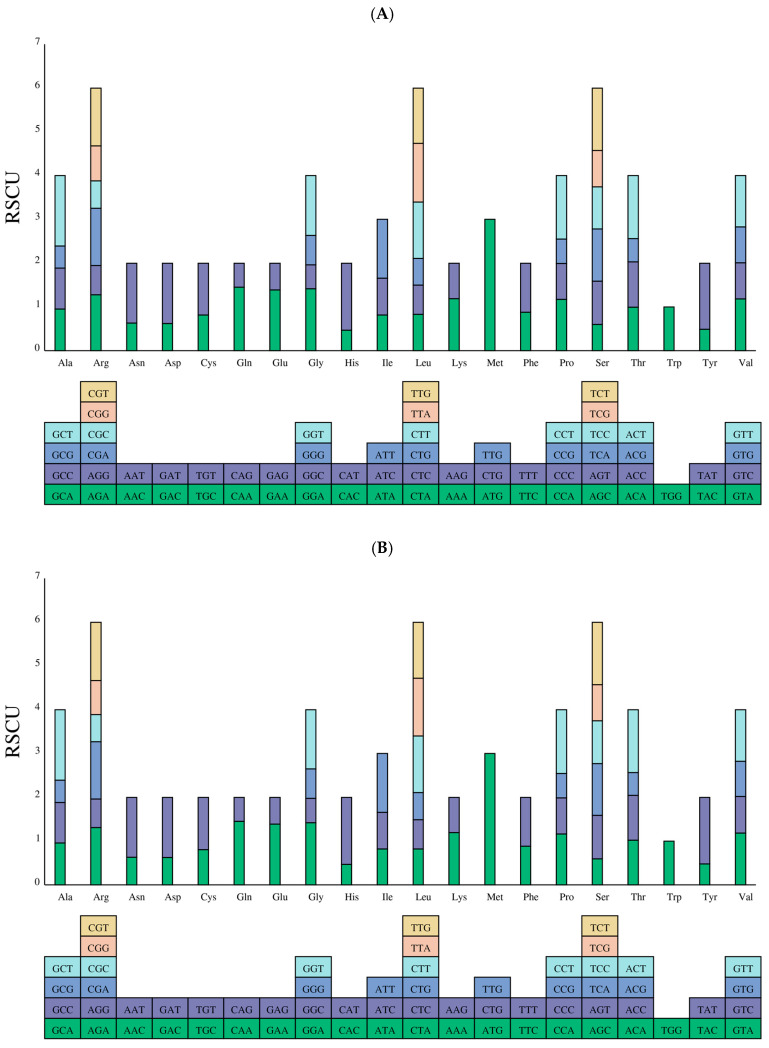
Relative synonymous codon usage (RSCU) in the mitochondrial genome of *D. nobile* Lindl. (**A**) and *D. denneanum* Kerr. (**B**). The *x*-axis represents the different kinds of amino acids. The *y*-axis represents the value of RSCU. The boxes below represent all the codons that encode each amino acid.

The results showed that all genes were encoded by 8768 and 8838 codons, which encode 20 amino acids in *D. nobile* Lindl. and *D. denneanum* Kerr., respectively (Supplementary Table S3). Notably, 5368 and 5548 codons had RSCU values greater than 1.0, indicating a higher frequency of usage. Furthermore, the RSCU values of UAA and UGA were greater than 1.0 among the three stop codons (UAA, UGA, UAG), indicating a higher frequency of usage in both species. All codons with an RSCU value greater than 1.0 ended with an A or U base (Figure 3).

### 2.4. Repeat Sequence of Mitochondrial Genomes in D. nobile Lindl. and D. denneanum Kerr.

**Figure 4:**
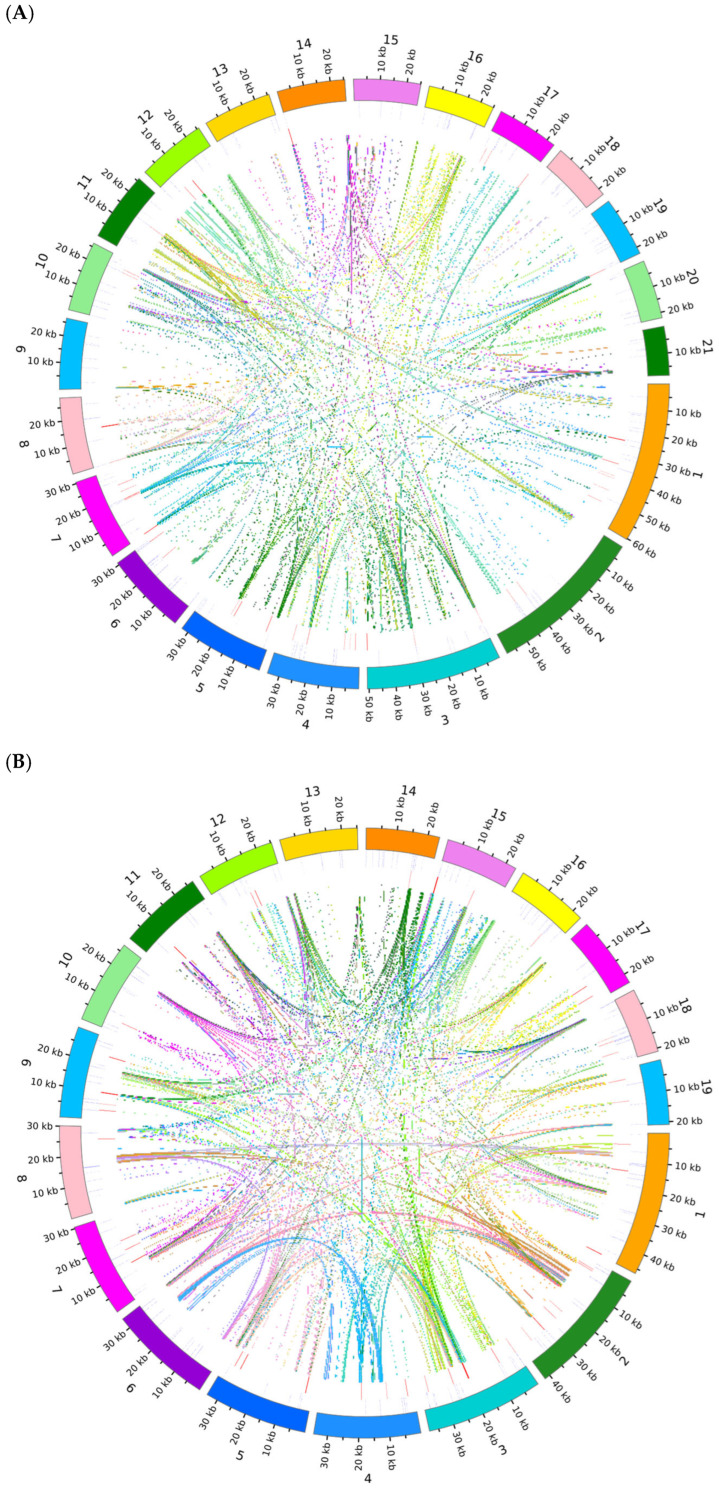
Repeat sequence distribution in the mitochondrial genomes of *D. nobile* Lindl. (**A**) and *D. denneanum* Kerr. (**B**). The outermost circle is the chromosomal sequence, followed by the SSRs and tandem repeat sequences, and the innermost is the dispersed repeat sequences. These are shown in different colors for each chromosome.

A total of 993 and 1491 repeat sequences were detected in the mitochondrial genomes of *D. nobile* Lindl. and *D. denneanum* Kerr., including 791 and 1283 dispersed repeats, 158 and 157 simple sequence repeats (SSRs), and 44 and 51 tandem repeats respectively (Figure 4). The dispersed repeats consisted of 385 and 574 forward repeats and 406 and 709 palindromic repeats; the maximum length of forward repeats was 887 bp and 981 bp, respectively, while the largest palindromic repeats had sizes of 709 bp and 939 bp. The total length of the dispersed repeats was 67,722 bp and 127,226 bp, accounting for 10.56% and 22.77% of the total mitochondrial genome length in both species (Supplementary Table S4). Notably, among the 158 and 157 SSRs, 43 and 33 mononucleotide repeats, 34 and 42 dinucleotide repeats, 22 and 22 trinucleotide repeats, 55 and 43 tetranucleotide repeats, 2 and 10 pentanucleotide repeats, and 2 and 7 hexanucleotide repeat types were identified, respectively (Supplementary Tables S5 and S6). Moreover, among the mononucleotide repeats, A/T repeats were the most common. The length of tandem repeats ranged from 12 bp to 60 bp, and their matching degree exceeded 66.0%. Additionally, the length ranged from 6 bp to 34 bp, with a matching degree exceeding 67.0%. Meanwhile, tandem repeats of *D. nobile* Lindl. were most frequently distributed on chromosome 1 (7 repeats), chr4 (5 repeats), and chr7 (6 repeats), whereas in *D. denneanum* Kerr., they were most frequent on chr1 (6 repeats), chr 2 (7 repeats), chr 3 (6 repeats), and chr 9 (5 repeats) (Supplementary Tables S7 and S8).

### 2.5. Substitution Rates of PCGs

**Figure 5:**
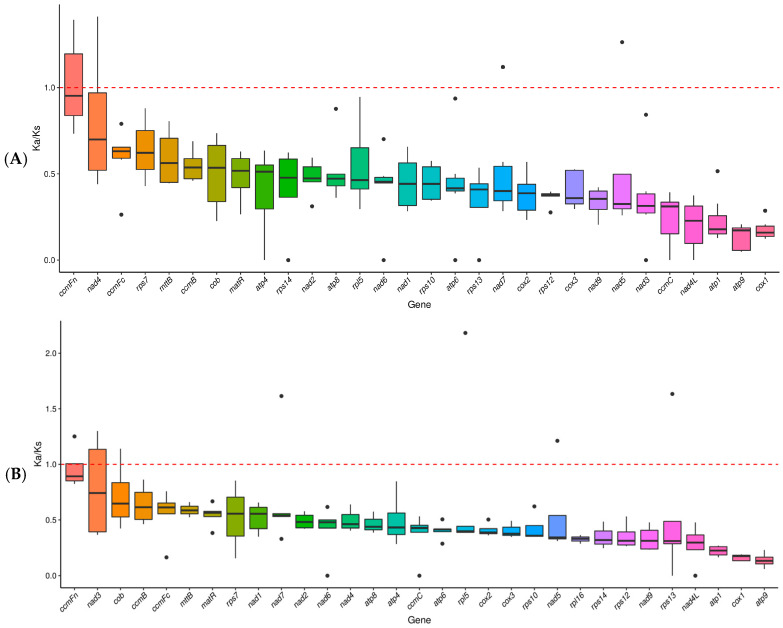
Boxplot of pairwise Ka/Ks values of protein-coding genes (PCGs) in the mitochondrial genomes among *D. nobile* Lindl. with different species, namely *D. amplum*, *Salvia miltiorrhiza*, *Nelumbo nucifera*, *Platycodon grandifloras*, *Senna tora*, and *Rhododendron simsii* (**A**), and *D. denneanum* Kerr. with different species, namely *D. amplum*, *Nicotiana tabacum*, *Ziziphus jujuba*, *Cocos nucifera*, and *Schisandra sphenanthera* (**B**). The *x*-axis indicates the different PCGs and the *y*-axis indicates the Ka/Ks values. Black dots represent outliners.

In this study, the ratio of the nonsynonymous to synonymous substitution rate (Ka/Ks) was calculated for 30 and 31 PCGs from the mitochondrial genomes of *D. nobile* Lindl. and *D. denneanum* Kerr., respectively, and compared to the mitochondrial genomes of other representative medicinal plants, in order to investigate the evolutionary rates of mitochondrial genes. As shown in Figure 5, the average Ka/Ks ratios were less than 1 in 29 PCGs of *D. nobile* Lindl. and all 31 PCGs of *D. denneanum* Lindl., indicating that these PCGs were subject to negative selection (purifying selection) to varying degrees during the evolutionary process, reflecting their high conservation. In contrast, the average Ka/Ks ratio of the *ccmFn* (1.017) gene in *D. nobile* Lindl. mitogenome was close to 1.0, indicating that it underwent neutral evolution (Supplementary Table S9).

### 2.6. DNA Transfer from Chloroplast to Mitochondrional Genome

**Figure 6:**
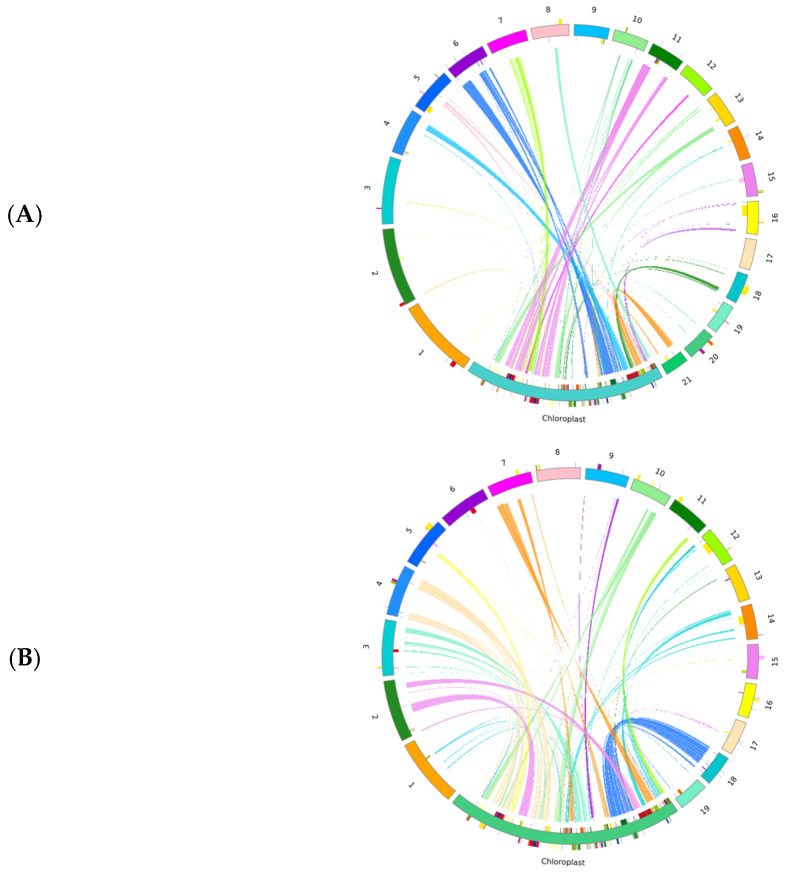
The visualization of homologous sequences between the chloroplast and mitochondrial genomes of *D. nobile* Lindl. (**A**) and *D. denneanum* Kerr. (**B**). The large outer arcs represent chromosome sequences. “Chloroplast” refers to the chloroplast genome, while the remaining parts represent the mitochondrial genome. The colored small arcs on the large arcs indicate different types of genes within each chromosome. The connecting lines in the middle show the homologous sequences between the chloroplast and mitochondrial genomes. Homologous sequences between different chromosomes within the chloroplast and mitochondrial genomes are represented by different colors.

Sequence similarity analysis revealed that there were 26 and 36 homologous fragments shared between the mitochondrial and chloroplast genomes, with total lengths of 32,657 bp and 27,558 bp, accounting for 5.09% and 4.93% of the total lengths of the mitogenomes of the two species, respectively (Figure 6). Among these, 9 and 11 fragments were longer than 1000 bp, with the longest fragments measuring 9550 bp and 3260 bp, and the shortest fragments (Chr 6, Chr14) being 29 bp and 73 bp respectively. As shown in Table 4, annotation of these homologous sequences identified 18 integrated chloroplast-derived genes in *D. nobile* Lindl. and 23 in *D. denneanum* Kerr. Moreover, two incomplete rRNA genes were also discovered in *D. nobile* Lindl.—*rrn18* (partial: 43.36%) and *rrn26* (partial: 2.83%)—as well as the *rpl16* gene and the incomplete rRNA gene *rrn26* (partial: 2.83%) in *D. denneanum* Kerr. It is noteworthy that all the transferred genes were tRNA genes and partial rRNA genes; no PCGs were found.

### 2.7. Synteny Analysis of D. nobile Lindl. and D. denneanum Kerr.

**Figure 7:**
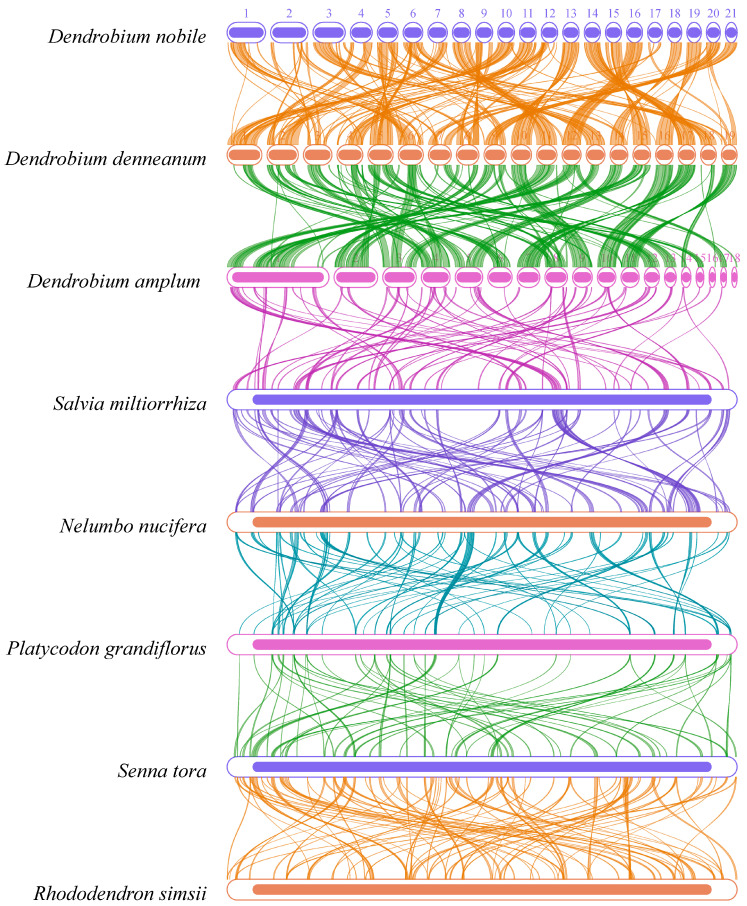
A synteny map comparing mitochondrial genomes of the different species (*D. nobile*, Lindl., *D. denneanum* Kerr., *D. amplum*, *S. miltiorrhiza*, *N. nucifera*, *P. grandiflorus*, *S. tora*, *R. simsii*). The boxes in each row represent the mitochondrial genomes, and the lines between every two mitochondrial genomes in the middle represent homologous regions.

**Figure 8:**
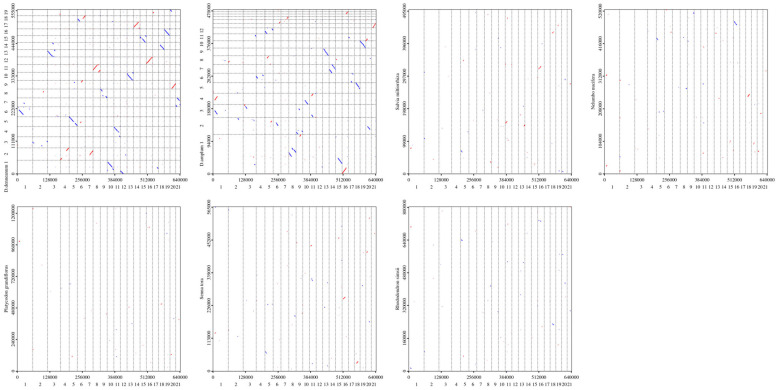
The dot plots showing the mitochondrial genome of the target species (*D. nobile* Lindl.) compared to that of other species (*D. denneanum* Kerr., *D. amplum*, *S. miltiorrhiza*, *N. nucifera*, *P. grandiflorus*, *S.tora*, *R. simsii*). The *x*-axis in each box represents the coordinates of the chromosomes in the target genome; the *y*-axis represents the coordinates of chromosomes from other species. The red line in each box indicates a forward comparison, whereas the blue line represents a reverse complementary comparison.

Based on sequence similarity analysis, a multiple synteny map was constructed for *D. nobile* Lindl., *D. denneanum* Kerr. and six other species (*D. amplum*, *Salvia miltiorrhiza*, *Nelumbo nucifera*, *Platycodon grandiflorus*, *Senna tora*, *Rhododendron simsii*) (Figure 7). A large number of syntenic collinear blocks were found between *D. nobile* Lindl. and *D. denneanum* Kerr. Meanwhile, analysis of the dot plot (Figure 8) revealed scattered syntenic regions between the mitochondrial genomes of the target species and the other five species (*S. miltiorrhiza*, *N. nucifera*, *P. grandiflorus*, *C. obtusifolia*, *R. simsii*), indicating a low degree of collinearity. Notably, collinearity analysis revealed that the syntenic blocks were not fully identical among *D. nobile* Lindl., *D. denneanum* Kerr., and *D. amplum*, indicating that the mitochondrial genomes of *Dendrobium* species have undergone extensive rearrangement events, leading to highly divergent structures and low conservation.

### 2.8. Phylogenetic Analysis of D. nobile Lindl. and D. denneanum Kerr.

**Figure 9:**
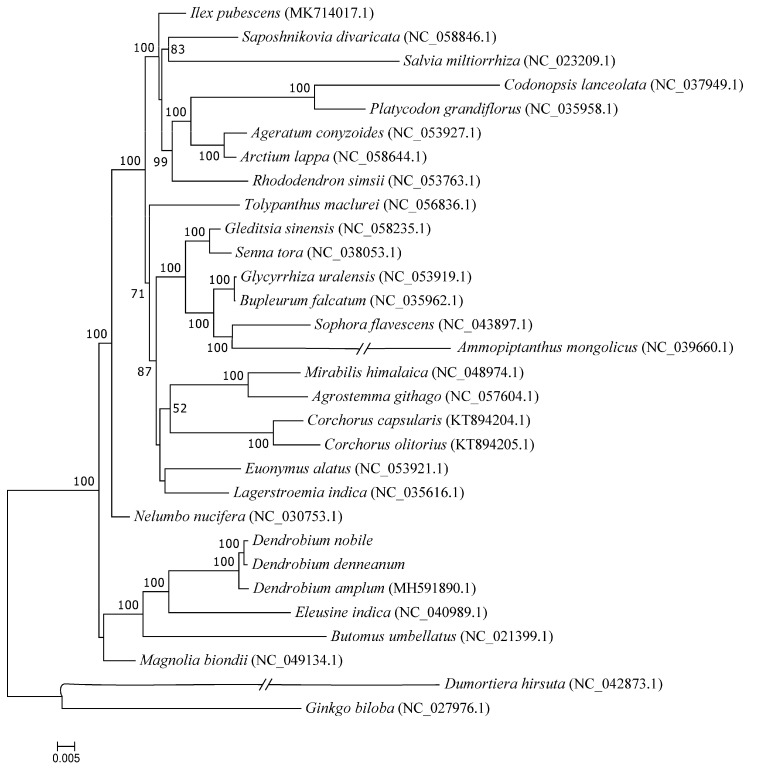
The phylogenetic tree was constructed based on mitochondrial genome CDS sequences from 30 representative traditional Chinese medicinal plants, namely: *D. nobile* Lindl., *D. denneanum* Kerr., *D. amplumz*, *L. indica*, *S. divaricate*, *A. lappa*, *G. sinensis*, *A. githago*, *T. maclurei*, *A. conyzoides*, *E. alatus*, *G. uralensis*, *R. simsii*, *M. biondii*, *M. himalaica*, *S. flavescens*, *D. hirsute*, *E. indica*, *A. mongolicus*, *S. tora*, *C. lanceolata*, *B. falcatum*, *P. grandifloras*, *N. nucifera*, *G. biloba*, *S. miltiorrhiza*, *B. umbellatus*, *I. pubescens*, *C. olitorius*, *C. capsularis*. The branch support was determined by computing 1000 non-parametric bootstrap replicates.

To determine the phylogenetic positions of *D. nobile* Lindl. and *D. denneanum* Kerr., phylogenetic trees were constructed using mitochondrial genome CDS sequences retrieved from GenBank for these *Dendrobium* species and other traditional medicinal plants (Figure 9). The results showed that the two target species, *D. nobile* Lindl. and *D. denneanum* Kerr., clustered together and further grouped with *D. amplum*. This suggests that the three *Dendrobium* species, which are placed in the same genus in traditional taxonomy, also exhibit a close molecular phylogenetic relationship. Molecular phylogenetic analysis thus supports the reliability of traditional classification and provides robust evidence for the close relationship among the three species distributed in southwestern China. In addition, species of the genus *Dendrobium* and *Eleusine indica* (Poaceae) formed another clade, which subsequently clustered with *Butomus umbellatus* (Butomaceae), and this larger clade further clustered with *Magnolia biondii* (Magnoliaceae), with 100% bootstrap, indicating a close phylogenetic relationship among these taxa.

## 3. Discussion

### 3.1. Characteristics of Complex Multi-Chromosome Circular Structures

Previous studies have revealed that the diversity in plant mitochondrial genome structure is a remarkable feature. For instance, species such as *Camellia sinensis* [22] and *Eucalyptus grandis* [23] possess a single circular structure, whereas *Cucumis sativus* [24] has been found to have three independent circular chromosomes. Similarly, the mitochondrial genome architecture in *Dendrobium* also displays both monocyclic and polycyclic structural types. For example, the mitochondrial genome of root tip cells from tissue-cultured *D. hancockii* [17] was meticulously assembled into one single large circular molecule, whereas most species in the genus *Dendrobium* exhibit a complex multibranched and multi-chromosomal conformation [18,19,20].

Our assembly results showed that the mitochondrial genomes of *D. nobile* Lindl. and *D. denneanum* Kerr. also exhibited a multi-chromosomal circular structure, with 21 and 19 contigs, respectively. Previous studies have extensively investigated both the monomeric circular and multi-chromosomal structures of mitochondrial genomes. Researchers have proposed that a single circular genomic map does not necessarily reflect the absolute in vivo conformation of the mitochondrial genome, since alternative configurations could be generated via intramolecular or intermolecular recombination [25]. Likewise, multi-chromosomal structures may be caused by environment-induced alterations of the mitochondrial genome. For instance, under stressful conditions, plants may downregulate the expression of genes that normally suppress recombination among short repetitive sequences in the mitochondrial genome [17]. In conclusion, the multi-chromosomal circular architecture of the mitochondrial genome contributes to the genomic complexity of *Dendrobium* species, and the underlying regulatory mechanism remains to be further elucidated.

### 3.2. The Phenomenon of Gene Loss

The composition of plant mitochondrial-encoded genes and the fate of lost genes have attracted considerable attention in previous studies [14]. Gene loss in the mitochondrial genomes of *Dendrobium* species has also been documented [19]. In the present study, we detected gene loss events in two species: *D. nobile* Lindl. and *D. denneanum* Kerr. Compared with the previously reported *D. nobile* Lindl., both species lost several non-core ribosomal protein genes, including *rps1*, *rps2*, *rps3*, *rps4*, *rps11*, and *rps19*. In addition, the previously reported *D. nobile* Lindl. loses an additional large ribosomal subunit gene, rpl16 [20]. Interestingly, gene loss patterns varied, even within the same species, according to geographical origin. Loss of the *rpl16* gene has been reported in other plants [26]. However, the concurrent loss of six *rps* genes encoding small ribosomal subunits in these two *Dendrobium* species is relatively rare. Such extensive gene loss may imply that the mitochondrial genomes of *Dendrobium* have undergone rapid structural evolution.

Similarly, the succinate dehydrogenase subunit genes *sdh3* and *sdh4* were not annotated in either species. These two genes have been lost repeatedly in the mitochondrial genomes of numerous other angiosperms [27] and are also commonly absent in Orchidaceae, consistent with the pattern observed in most monocots [28,29]. In fact, mitochondrial gene loss represents a widespread and ongoing process in angiosperms. As suggested by previous studies, most lost mitochondrial genes may have been transferred to the nuclear genome via endosymbiotic gene transfer [30,31]. Like other orchids, *Dendrobium* species may have rewired their protein synthesis and energy metabolism pathways. Such modifications could potentially compensate for mitochondrial gene loss through alternative pathways or mechanisms, thereby conferring adaptive advantages under specific environmental conditions [28]. With the increasing availability of genomic data for *Dendrobium* species, further investigation may uncover the reasons behind these genetic patterns.

### 3.3. Abundant Repeat Sequences as Potentially Important Molecular Markers

Previous studies have shown that plant mitochondrial genomes contain numerous repeat sequences, which represent potentially important molecular markers [32] and can be used in genetic and evolutionary research [33]. Furthermore, no clear correlation has been observed between mitochondrial genome size and the number of repeat sequences [34,35]. Our results are consistent with these reports: the complete mitochondrial genomes of *D. nobile* Lindl. and *D. denneanum* Kerr. were 641,414 bp and 558,760 bp in length, respectively, while 993 and 1491 repeat sequences were identified in the two species. In addition, SSRs are frequently employed in genetic diversity assessment [36,37,38]. In this study, 158 and 157 SSRs were detected within the mitochondrial genomes of the two species, respectively, providing abundant potential molecular markers for future species identification. Among these SSRs, mononucleotide A/T repeats were the most abundant, similar to observations in other *Orchidaceae* species [35,39]. This may be attributed to the lower binding energy required to break A-T bonds compared with G-C bonds [40,41]. Meanwhile, 791 and 1283 dispersed repeats, as well as 44 and 51 tandem repeats, were identified, respectively, and tandem repeats were found to be unevenly distributed across the mitochondrial genomes of the two *Dendrobium* species. As previous studies have suggested, diverse repetitive sequences can mediate frequent recombination events within the mitochondrial genome [42]. During evolution, frequent recombination may have played a key role in reshaping the mitochondrial genome structure and conformation, leading to increased complexity [43]. Furthermore, recombination can induce genomic rearrangements, which may affect gene function and enhance the intrinsic adaptability of plants [44,45].

### 3.4. Intracellular Gene Transfer

As is well known, a prominent characteristic of plant mitochondrial genome evolution is the frequent integration of foreign DNA via horizontal gene transfer [46]. Therefore, investigating gene transfer events is critical for exploring the evolutionary dynamics of plant mitochondrial genomes. Sequence transfers between the chloroplast and mitochondrial genomes are generally referred to as intracellular gene transfer (IGT). The transfer of tRNA gene sequences from the chloroplast to the mitochondrion is common in flowering plants [47]. Our study revealed that 18 of the 29 tRNA genes in *D. nobile* Lindl. and 23 of the 33 tRNA genes in *D. denneanum* Kerr. were derived from the chloroplast genome, indicating high conservation of chloroplast tRNA genes in the two *Dendrobium* species. The homologous fragments between the mitochondrial and chloroplast genomes accounted for 5.09% and 4.93% of the mitochondrial genomes in the two species, respectively. These values were lower than the range (6.81–10.34%) reported in other Orchidaceae species [39,48], but similar to the approximate 5% observed in most other angiosperms [25]. Previous studies have proposed that intergenomic transfer is the primary cause of the abundant tRNAs in the genus *Dendrobium* [20], reflecting the conservation of transport functions in the mitochondrial genome. This pattern is also widespread in angiosperms [49]. To date, the molecular mechanism underlying IGT remains unclear, and such events have been suggested to be largely random and independent [50]. With more mitochondrial genomic data becoming available for *Dendrobium* species, the mechanisms of IGT may be further elucidated. Elucidating the patterns of inter-organellar sequence transfer is also of great significance for tracing ancient recombination events and structural variations in plant mitochondrial genomes, warranting further in-depth investigation.

## 4. Materials and Methods

### 4.1. Plant Materials

Seedlings of *D. nobile* Lindl. and *D. denneanum* Kerr. were collected from imitation-wild cultivated populations growing in Chishui City, Guizhou Province, and Guangyuan City, Sichuan Province, in southwestern China. The GPS coordinates of the two populations were recorded as E105°52′, N28°30′ and E105°18′, N32°14′. Young, fresh, healthy and disease-free leaves were excised from seedlings and immediately stored in self-sealing bags containing dry silica gel. They were then taken back to the laboratory and stored in a refrigerator at −80 °C prior to use. The collected species were authenticated by the authors. Finally, these accessions were sent to Genepioneer Biotechnologies Co., Ltd. (Nanjing, China) for sequencing.

### 4.2. DNA Extraction and Sequencing

Total genomic DNA was extracted from fresh leaves of *Dendrobium* species using a modified CTAB method [51]. DNA integrity was detected by electrophoresis on 1.0% (*w*/*v*) agarose gel. DNA concentration and purity were quantified using a NanoDrop One spectrophotometer (Thermo Fisher Scientific, Waltham, MA, USA). Only DNA samples with an A260/A280 ratio of 1.8–2.0 and an A260/A230 ratio of 2.0–2.2, indicating high purity without protein or carbohydrate contamination, were retained for library construction. The electrophoretic bands were clear and distinct, with no signs of diffusion or tailing and the assessment grade of DNA quality was A, indicating the DNA samples met the quality requirements.

To achieve high-accuracy, full-length mitochondrial genome assembly, short-read and long-read sequencing technologies were combined in this study. The short-read sequencing platform was Illumina Novaseq 6000 (Illumina, San Diego, CA, USA) and the paired-end sequencing (PE) read length was 150 bp, and fastp (version 0.23.4) software was used to filter the original data and get high-quality reads. The long-read sequencing platform was Nanopore PromethION (Nanopore, Oxford, UK); then, the sequencing data was filtered by filtlong (v0.2.1) software with the parameters: —min_length 1000—min_mean_q 7.

### 4.3. Assembly and Annotation

Plant mitochondrial genes are very conserved. Taking advantage of this feature, the comparison software Minimap2 (v2.1) [52] was used to compare the original long-read sequencing data with the reference gene sequence (plant mitochondrial core gene, https://github.com/xul962464/plant_mt_ref_gene, accessed on 10 April 2023); sequences with similar fragments longer than 50 bp were selected as candidate sequences. The candidate sequences with more aligned genes (one sequence contains multiple core genes) and higher alignment quality (covering more complete core genes) were selected as the seed sequence. Then, compared the original long-read sequencing data with the seed sequence, the sequences with minimum overlap of 1kb and at least 70% similarity were added to the seed sequence and the original data was iteratively aligned to the seed sequence, so as to obtain all long-read sequencing data for the mitochondrial genome. Then, the assembly software canu [53] was used to correct the long-read sequencing data obtained, and bowtie2 (v2.3.5.1) was used to align the short-read sequencing data to the corrected sequence. Then, the default parameter Unicycler (v0.4.8) was used to compare the above short-read sequencing data and the corrected long-read sequencing data for concatenation. Finally, the ring mitochondrial genome of *D. nobile* Lindl. and *D. denneanum* Kerr. was obtained.

Mitochondrial genome structure annotation was divided into the following steps: (1) For the encoding protein and rRNA, BLAST v2.10.1 was used to align the published plant mitochondrial sequences as refs; further manual adjustments were made for related species. (2) tRNA was annotated using tRNAs-canSE (http://lowelab.ucsc.edu/tRNAscan-SE/, accessed on 12 April 2023) [54]. (3) The Open Reading Frame (ORF) Finder (http://www.ncbi.nlm.nih.gov/gorf/gorf.html, accessed on 12 April 2023) was used to annotate ORFs; the shortest length was set to 102 bp and redundant sequences and sequences with overlap with known genes were excluded. Sequence alignments greater than 300 bp were annotated to the NR library. To obtain more accurate annotation results, the above results were checked and manually corrected. Then, the mitochondrial genome was mapped using OGDRAW (https://chlorobox.mpimp-golm.mpg.de/OGDraw.html, accessed on 12 April 2023).

All assembly mitochondrial genome sequences of *D. nobile* Lindl. and *D. denneanum* Kerr. have been deposited in GenBank and assigned accession numbers: *D. nobile* Lindl.: PX171404-PX171424, *D. denneanum* Kerr.: PX171425-PX171443 (Table 1).

### 4.4. RNA Editing Sites

In higher plants, RNA editing is a post-transcriptional process essential for mitochondrial gene expression [55]. The plant mitochondrial gene-encoding proteins were used as the reference proteins to identify RNA editing sites within the mitochondrial genes of both species. In this study, the mitochondrial genome RNA editing site was predicted according to the tool PmtREP (http://112.86.217.82:9919/#/tool/alltool/detail/336, accessed on 14 April 2023), constructed by Genepioneer Biotechnologies Co., Ltd. (Nanjing, China).

### 4.5. RSCU Analysis

CodonW1.4.4 was used to employ RSCU to assess synonymous codon usage patterns in the mitochondrial genome [56]. The R package ggplot2 (v3.5.1) was used to generate visualizations of the RSCU data, providing a clear and informative representation of codon usage patterns. Generally, an RSCU value greater than 1.0 indicates a relatively high usage frequency and a preference for specific amino acid codons; an RSCU value equal to 1.0 indicates no usage bias; and an RSCU value less than 1.0 indicates a low usage frequency.

### 4.6. Repeat Sequences

Three kinds of repeats (simple sequence, tandem, and dispersed) were detected in the *Dendrobium* species mitochondrial genome. The MIcroSAtellite (MISA) identification tool Perl script was used to detect simple sequence repeats [57] (v1.0, parameter: 1-10 2-5 3-4 4-3 5-3 6-3). Tandem repeats (>6 bp repeat units) were detected using Tandem Repeats Finder v4.09 software (http://tandem.bu.edu/trf/trf.submit.options.html, accessed on 13 April 2023) (trf409.linux64, parameter: 2 7 7 80 10 50 2000 -f -d -m) [58] with default parameters. Dispersed repeats were detected using blastn (v2.10.1, parameters: -word size 7, evalue 1 × 10^−5^, remove redundancy, remove tandem duplication). Circos v0.69-5 was used to visualize these repeats.

### 4.7. The Non-Synonymous (Ka) and Synonymous (Ks) Value

To understand the natural selection pressure in the evolution of the genus *Dendrobium*, homologous protein sequences of *D. nobile* Lindl., *D. denneanum* Kerr. and other species, including *D. amplum* (MH591890.1), *S. miltiorrhiza* (NC023209.1), *N. nucifera* (NC030753.1), *P. grandifloras* (NC035958.1), *S. tora* (NC038053.1), *R. simsii* (NC053763.1), *N.* (NC006581.1), *Z. jujuba* (NC029809.1), *C. nucifera* (NC031696.1), and *S. sphenanthera* (NC042758.1), were obtained using BLASTN v2.10.1. Then, the shared protein-coding genes were aligned using MAFFT v7 [59]. Ka/Ks ratios were calculated using KaKs_Calculator v 2.0 [60]. If Ka/Ks > 1.0, positive selection is inferred; if Ka/Ks = 1.0, neutral selection is assumed; and if Ka/Ks < 1.0, purifying selection is indicated.

### 4.8. Homologous Sequences Between Mitochondrial and Chloroplast Genome

The homologous sequences between the mitochondrial genome and chloroplast (SRR35031937, SRR35031936) were aligned using BLAST v2.10.1 software with a similarity threshold of no less than 70%. The homologous fragments between mitochondrial and chloroplast genomes were visually displayed using the Circos v0.69-5 procedure.

### 4.9. Synteny Analysis

Synteny analysis was conducted by comparing whole-genome alignments between the assembled sequence and selected sequences of other related species, resulting in a dot plot. The assembled species and selected species were compared pairwise, and collinearity maps were generated.

### 4.10. Phylogenetic Tree

To analyze the phylogenetic status of *Dendrobium* plants, the complete mitochondrial genomes of 28 other traditional Chinese medicinal plants were downloaded from the NCBI database, namely: *D. amplumz* (MH591890.1), *Lagerstroemia indica* (NC035616.1), *Saposhnikovia divaricate* (NC058846.1), *Arctium lappa* (NC058644.1), *Gleditsia sinensis* (NC058235.1), *Agrostemma githago* (NC057604.1), *Tolypanthus maclurei* (NC056836.1), *Ageratum conyzoides* (NC053927.1), *Euonymus alatus* (NC053921.1), *Glycyrrhiza uralensis* (NC053919.1), *R. simsii* (NC053763.1), *Magnolia biondii* (NC049134.1), *Mirabilis himalaica* (NC048974.1), *Sophora flavescens* (NC043897.1), *Dumortiera hirsute* (NC042873.1), *Eleusine indica* (NC040989.1), *Ammopiptanthus mongolicus* (NC039660.1), *S. tora* (NC038053.1), *Codonopsis lanceolata* (NC037949.1), *Bupleurum falcatum* (NC035962.1), *P. grandifloras* (NC035958.1), *N. nucifera* (NC030753.1), *Ginkgo biloba* (NC027976.1), *S. miltiorrhiza* (NC023209.1), *Butomus umbellatus* (NC021399.1), *Ilex pubescens* (MK714017.1), *Corchorus olitorius* (KT894205.1), *Corchorus capsularis* (KT894204.1). The shared CDSs of *Dendrobium* species’ protein-coding genes were aligned using the MAFFT [59] procedure. Maximum likelihood (ML): a maximum likelihood (ML) phylogenetic tree was constructed by RAxML v8.2.10 (https://cme.h-its.org/exelixis/software.html, accessed on 15 April 2023) (GTRGAMMA model) estimation with 1000 bootstrap replications. Bayesian inference (BI): The optimal nucleotide substitution model was calculated by jModelTest v2.1.10 (https://github.com/ddarriba/jmodeltest2, accessed on 15 April 2023), and then MrBayes v3.2.7a (http://nbisweden.github.io/MrBayes/, accessed on 15 April 2023) was used to establish a Bayesian inference (BI) phylogenetic tree; the parameters of MrBayes v3.2.7 software were based on jModelTest v2.1.10 results.

## 5. Conclusions

In this study, the complete mitochondrial genome of *D. denneanum* Kerr. was the first to be reported. The results revealed that the mitochondrial genomes of *D. nobile* Lindl. and *D. denneanum* Kerr. had typical complex multi-chromosome circular structures. Furthermore, it was found that the two species possessed abundant repeat sequences as potentially important molecular markers and simultaneous loss of most *rps* genes encoding small ribosomal proteins. A total of 26 and 36 chloroplast genomic DNA fragments were transferred to the mitochondrial genome of the two species, indicating that most tRNA genes had undergone IGT. Additionally, synteny and phylogenetic analyses were also conducted, enhancing our understanding of evolutionary relationships and genetic models. In conclusion, this study lays the foundation for further research on molecular markers, species identification, and evolutionary relationships among these species. It also contributes to understanding how mitochondrial genomes adapt to environmental pressures for survival.

## Figures and Tables

**Table 1:** Assembly results of mitochondrial genomes in *D. nobile* Lindl. and *D. denneanum* Kerr.

Sequence Name	Accession Number (NCBI Public Database)	Sequence Type	Genome Length (bp)	GC Content (%)
*D. nobile-1*	PX171404	circular	60,351	44.34
*D. nobile-2*	PX171405	circular	58,650	42.12
*D. nobile-3*	PX171406	circular	50,648	44.38
*D. nobile-4*	PX171407	circular	34,419	41.66
*D. nobile-5*	PX171408	circular	32,628	43.61
*D. nobile-6*	PX171409	circular	30,873	39.64
*D. nobile-7*	PX171410	circular	30,500	41.39
*D. nobile-8*	PX171411	circular	28,639	43.52
*D. nobile-9*	PX171412	circular	26,558	44.76
*D. nobile-10*	PX171413	circular	26,352	43.83
*D. nobile-11*	PX171414	circular	26,079	44.23
*D. nobile-12*	PX171415	circular	25,795	40.64
*D. nobile-13*	PX171416	circular	25,661	42.91
*D. nobile-14*	PX171417	circular	25,465	44.54
*D. nobile-15*	PX171418	circular	25,109	44.98
*D. nobile-16*	PX171419	circular	25,010	45.95
*D. nobile-17*	PX171420	circular	23,203	43.35
*D. nobile-18*	PX171421	circular	22,578	43.99
*D. nobile-19*	PX171422	circular	22,544	45.29
*D. nobile-20*	PX171423	circular	22,045	46.18
*D. nobile-21*	PX171424	circular	18,307	41.46
*D. nobile*_Total			641,414	43.40
*D. denneanum-1*	PX171425	circular	46,536	42.48
*D. denneanum-2*	PX171426	circular	41,831	41.20
*D. denneanum-3*	PX171427	circular	38,036	43.27
*D. denneanum-4*	PX171428	circular	34,489	44.77
*D. denneanum-5*	PX171429	circular	33,660	44.68
*D. denneanum-6*	PX171430	circular	33,588	44.41
*D. denneanum-7*	PX171431	circular	30,484	42.13
*D. denneanum-8*	PX171432	circular	30,126	43.26
*D. denneanum-9*	PX171433	circular	29,441	44.87
*D. denneanum-10*	PX171434	circular	27,262	42.94
*D. denneanum-11*	PX171435	circular	27,213	43.28
*D. denneanum-12*	PX171436	circular	25,272	44.87
*D. denneanum-13*	PX171437	circular	25,190	43.94
*D. denneanum-14*	PX171438	circular	23,734	44.46
*D. denneanum-15*	PX171439	circular	23,490	44.81
*D. denneanum-16*	PX171440	circular	23,316	45.07
*D. denneanum-17*	PX171441	circular	23,101	44.01
*D. denneanum-18*	PX171442	circular	21,064	37.99
*D. denneanum-19*	PX171443	circular	20,927	44.88
*D. denneanum*_Total			558,760	43.49

**Table 2:** Gene composition in the mitochondrial genomes of *D. nobile* Lindl. and *D. denneanum* Kerr.

Group of Genes	*D. nobile* Lindl.	*D. denneanum* Kerr.
	Name of Genes	Name of Genes
ATP synthase	*atp1*, *atp4*, *atp6*, *atp8*, *atp9*	*atp1*, *atp4*, *atp6*, *atp8*, *atp9*
Cytochrome c biogenesis	*ccmB*, *ccmC*, *ccmFc* *, *ccmFn*	*ccmB*, *ccmC*, *ccmFc* *, *ccmFn*
Ubiquinol cytochrome c reductase	*cob*	*cob*
Cytochrome c oxidase	*cox1*, *cox2* **, *cox3*	*cox1*, *cox2* **, *cox3*
Maturases	*matR*	*matR*
Transport membrane protein	*mttB*	*mttB*
NADH dehydrogenase	*nad1* ****, *nad2* ****, *nad3*, *nad4* ***, *nad4L*, *nad5* ****, *nad6*, *nad7* ****, *nad9*	*nad1* ****, *nad2* ****, *nad3*, *nad4* ***, *nad4L*, *nad5* ****, *nad6*, *nad7* ****, *nad9*
Large subunit of ribosome	*rpl5*	*rpl16*, *rpl5*
Small subunit of ribosome	*rps10* *, *rps12*, *rps13*, *rps14*, *rps7*	*rps10* *, *rps12*, *rps13*, *rps14*, *rps7*
Ribosomal RNAs	*rrn18*, *rrn26*, *rrn5*	*rrn18*, *rrn26*, *rrn5*
Transfer RNAs	*trnC-GCA(2)*, *trnD-GTC(2)*, *trnE-TTC(2)*, *trnF-GAA*, *trnG-GCC*, *trnH-GTG*, *trnI-TAT* *, *trnK-TTT*, *trnL-TAG*, *trnM-CAT(4)*, *trnN-GTT(3)*, *trnQ-TTG(2)*, *trnR-ACG*, *trnR-TCT*, *trnS-GCT*, *trnS-GGA*, *trnT-TGT*, *trnV-GAC*, *trnY-GTA(2)*	*trnA-TGC* *, *trnC-GCA(3)*, *trnD-GTC(2) trnE-TTC(2)*, *trnF-GAA(2)*, *trnG-GCC*, *trnH-GTG(2)*, *trnK-TTT*, *trnL-TAG*, *trnM-CAT(5)*, *trnN-GTT(2)*, *trnP-TGG*, *trnQ-TTG*, *trnR-TCT*, *trnS-GCT(2)*, *trnS-GGA*, *trnT-GGT*, *trnT-TGT*, *trnV-GAC*, *trnW-CCA*, *trnY-GTA*

Notes: * indicates one intron, ** indicates two introns, *** indicates three introns, **** indicates four introns; Gene (2) indicates number of copies of multi-copy genes.

**Table 3:** Statistic results of change in the hydrophilic and hydrophobic properties induced by RNA editing in the mitochondrial genomes of *D. nobile* Lindl. and *D. denneanum* Kerr.

Type	RNA Edit	*D. nobile* Lindl.		*D. denneanum* Kerr.	
		No.	%	No.	%
hydrophilic–hydrophilic	CAC (H) => TAC (Y)	10		10	
	CAT (H) => TAT (Y)	19		18	
	CGC (R) => TGC (C)	12		13	
	CGT (R) => TGT (C)	32		32	
	total	73	13.30%	73	13.20%
hydrophilic–hydrophobic	ACA (T) => ATA (I)	5		5	
	ACG (T) => ATG (M)	7		8	
	ACT (T) => ATT (I)	5		5	
	CGG (R) => TGG (W)	34		34	
	TCA (S) => TTA (L)	78		78	
	TCC (S) => TTC (F)	37		38	
	TCG (S) => TTG (L)	42		43	
	TCT (S) => TTT (F)	56		54	
	ACC (T) => ATC (I)			1	
	total	264	48.09%	266	48.10%
hydrophilic–stop	CGA (R) => TGA (X)	2		2	
	total	2	0.36%	2	0.36%
hydrophobic–hydrophilic	CCA (P) => TCA (S)	9		9	
	CCC (P) => TCC (S)	14		14	
	CCG (P) => TCG (S)	7		7	
	CCT (P) => TCT (S)	20		20	
	total	50	9.11%	50	9.04%
hydrophobic–hydrophobic	CCA (P) => CTA (L)	48		48	
	CCC (P) => CTC (L)	10		10	
	CCC (P) => TTC (F)	6		6	
	CCG (P) => CTG (L)	27		28	
	CCT (P) => CTT (L)	28		29	
	CCT (P) => TTT (F)	14		14	
	CTC (L) => TTC (F)	6		5	
	CTT (L) => TTT (F)	11		12	
	GCA (A) => GTA (V)	1		1	
	GCC (A) => GTC (V)	1		1	
	GCG (A) => GTG (V)	4		4	
	GCT (A) => GTT (V)	4		4	
	total	160	29.14%	162	29.29%
	All	549	100%	553	100%

Note: Type indicates the type of hydrophilic/hydrophobic property change; RNA editing indicates the type of RNA editing; Number indicates the number of RNA edits; Percentage indicates the proportion.

**Table 4:** (A) DNA fragments transferred from the chloroplast to the mitochondrial genome in *D. nobile* Lindl. (B) DNA fragments transferred from the chloroplast to the mitochondrial genome in *D. denneanum* Kerr.

Subject-Mt	Identity (%)	Alignment Length (bp)	CP Start	CP End	Mt Start	Mt End	Genes
(**A**)							
chr6	98.942	9550	36,006	45,537	233	9752	*trnG-GCC*, *trnM-CAT*, *trnS-GGA*
chr6	95.881	1651	47,002	48,645	30,871	29,252	*trnF-GAA* (partial: 72.60%)
chr6	95.641	803	31,224	32,025	22,716	23,502	*trnY-GTA*, *trnE-TTC*
chr6	98.529	272	30,949	31,220	22,343	22,614	*trnD-GTC*
chr6	97.03	101	149,841	149,941	17,972	17,872	*trnH-GTG*
chr6	97.03	101	85,443	85,543	17,872	17,972	*trnH-GTG*
chr6	100	29	8330	8358	9530	9502	*trnS-GGA* (partial: 29.90%)
chr11	99.89	2738	131,483	134,220	8050	5313	*trnV-GAC*
chr11	99.89	2738	101,164	103,901	5313	8050	*trnV-GAC*
chr11	94.6	2315	108,462	110,754	26,078	23,809	*trnN-GTT*, *trnR-ACG*
chr11	94.6	2315	124,630	126,922	23,809	26,078	*trnN-GTT*, *trnR-ACG*
chr7	82.328	481	124,641	125,105	13,428	12,984	*trnN-GTT*
chr7	82.328	481	110,279	110,743	12,984	13,428	*trnN-GTT*
chr7	95.902	122	112,241	112,362	22,008	21,887	*trnL-TAG*
chr21	94.108	2529	10,344	12,860	5898	3455	*trnR-TCT*
chr10	95.756	1461	45,496	46,949	21,005	22,461	*trnT-TGT*
chr10	86.89	717	86,889	87,579	10,316	9640	*trnM-CAT*
chr10	86.89	717	147,805	148,495	9640	10,316	*trnM-CAT*
chr5	88.062	1089	6215	7264	18,153	19,184	*trnQ-TTG*
chr16	90.596	319	6828	7133	1	318	*trnQ-TTG*
chr14	96.25	80	110,362	110,441	10,364	10,285	*trnN-GTT*
chr14	96.25	80	124,943	125,022	10,285	10,364	*trnN-GTT*
chr2	74.183	887	132,017	132,880	1577	719	*rrn18* (partial: 43.36%)
chr2	74.183	887	102,504	103,367	719	1577	*rrn18* (partial: 43.36%)
chr1	85.567	97	128,685	128,781	7487	7391	*rrn26* (partial: 2.83%)
chr1	85.567	97	106,603	106,699	7391	7487	*rrn26* (partial: 2.83%)
		32,657					
(**B**)							
chr2	95.726	117	118,331	118,447	23,583	23,467	*trnL-TAG*
chr18	96.258	3260	42,829	46,072	4908	1705	*trnS-GGA*
chr18	97.666	2442	36,272	38,708	11,046	8629	*trnM-CAT*, *trnG-GCC*
chr18	92.341	1384	32,038	33,384	13,155	14,505	*trnT-GGT*
chr18	93.977	1129	46,066	47,182	1366	261	*trnT-TGT*
chr18	88.51	1436	47,578	48,981	21,064	19,719	*trnF-GAA* (partial: 72.60%)
chr18	96.243	346	31,094	31,431	12,390	12,735	*trnD-GTC*
chr18	97.487	199	31,659	31,857	12,756	12,954	*trnE-TTC*
chr10	98.754	883	139,318	140,199	19,389	18,516	*trnA-TGC*
chr10	98.754	883	107,181	108,062	18,516	19,389	*trnA-TGC*
chr7	96.226	159	10,336	10,493	30,326	30,484	*trnR-TCT*
chr14	96.355	1015	83,975	84,984	23,734	22,730	*rpl16*
chr14	95.89	73	157,239	157,311	7081	7009	*trnM-CAT* (partial: 98.65%)
chr14	95.89	73	90,069	90,141	7009	7081	*trnM-CAT* (partial: 98.65%)
chr4	99.157	1661	133,160	134,813	1922	262	*trnN-GTT*
chr4	99.157	1661	112,567	114,220	262	1922	*trnN-GTT*
chr4	97.806	1185	104,079	105,261	28,208	27,032	*trnV-GAC*
chr4	97.806	1185	142,119	143,301	27,032	28,208	*trnV-GAC*
chr4	91.701	1229	87,073	88,275	3828	5032	*trnH-GTG*
chr4	90.451	932	159,105	160,010	5032	4125	*trnH-GTG*
chr4	85.896	787	28,311	29,075	3792	3059	*trnC-GCA*
chr3	84.582	467	68,239	68,700	4951	5385	*trnP-TGG*
chr3	74.183	887	105,133	105,996	19,287	18,429	*rrn18* (partial: 43.36%)
chr3	74.183	887	141,384	142,247	18,429	19,287	*rrn18* (partial: 43.36%)
chr3	80.465	430	48,629	49,046	5421	5828	*trnF-GAA*
chr9	97.03	101	159,275	159,375	26,630	26,530	*trnH-GTG*
chr9	97.03	101	88,005	88,105	26,530	26,630	*trnH-GTG*
chr12	91.185	329	7007	7322	2256	2583	*trnQ-TTG*
chr1	85.042	722	157,239	157,929	33,021	32,351	*trnM-CAT*
chr1	85.042	722	89,451	90,141	32,351	33,021	*trnM-CAT*
chr19	94.353	425	8244	8664	20,513	20,927	*trnS-GCT*
chr19	78.409	88	45,483	45,569	20,864	20,777	*trnS-GCT*
chr17	96.386	83	112,990	113,072	13,714	13,632	*trnN-GTT*
chr17	96.386	83	134,308	134,390	13,632	13,714	*trnN-GTT*
chr6	86.598	97	109,232	109,328	22,946	22,850	*rrn26* (partial: 2.83%)
chr6	86.598	97	138,052	138,148	22,850	22,946	*rrn26* (partial: 2.83%)
		27,558					

## Data Availability

All assembled mitochondrial genome sequences of *D. nobile* Lindl. and *D. denneanum* Kerr. have been deposited in GenBank and assigned the following accession numbers: *D*. *nobile* Lindl.: PX171404-PX171424, *D*. *denneanum* Kerr.: PX171425-PX171443.

## Supplementary Materials

The following supporting information can be downloaded at: https://www.mdpi.com/article/10.3390/ijms27083441/s1.
